# Empowerment, Self-Management and Illness Perception of Users of an Online Self-Help Platform for Tinnitus: A Cross-Sectional Study

**DOI:** 10.3390/jcm15114043

**Published:** 2026-05-23

**Authors:** Jorge Piano Simões, Milena Engelke, Hazel Goedhart, Markku Vesala, Winfried Schlee, Steven Marcrum

**Affiliations:** 1 Department of Psychology, Health and Technology, University of Twente, 7522 NB Enschede, The Netherlands; 2 Department of Psychiatry and Psychotherapy, University of Regensburg, 93053 Regensburg, Germany; 3 Tinnitus Hub, 1071 LR Amsterdam, The Netherlands; 4 Institute for Information and Process Management, Eastern Switzerland University of Applied Sciences, 9471 Buchs, Switzerland; 5 Department of Otolaryngology, University Hospital Regensburg, 93053 Regensburg, Germany

**Keywords:** tinnitus, self-management, empowerment, perception, representation, self-help platform, chronic care

## Abstract

**Background:** Tinnitus is a common and potentially distressing phenomenon for which no broadly effective curative treatment exists. Self-management skills and empowerment are crucial for coping with chronic conditions, but empirical studies investigating the association of these on individuals burdened by tinnitus are scarce. The primary aim of this cross-sectional study was to investigate the association between the use of an online self-help platform for people with tinnitus and self-perceptions of empowerment, self-management skills, and the cognitive and emotional representations of tinnitus. **Methods:** One hundred and fifty-two adult participants were recruited from an online self-help platform for people with tinnitus, resulting in a self-selected convenience sample. Self-management skills were assessed using the Patient Assessment of Chronic Illness Care (PACIC) and the Partners in Health Questionnaire. The cognitive and emotional representations of tinnitus were measured with the Illness Perception Questionnaire. Finally, the Empowering Processes and Outcomes Questionnaire was used to evaluate empowerment associated with engagement in the self-help platform. The type and frequency of user activity on the self-help platform were used to explore the relationship between the nature of contributions to the platform and the measured outcomes. **Results:** The key findings include: (1) The representations of tinnitus were negatively related to their ability to self-manage the condition. (2) The duration of tinnitus did not correlate with improved self-management skills. (3) Comparing those who visited a healthcare provider for their tinnitus with those who did not, we found that treatment adherence was higher among participants with clinical visits. (4) Participants in this study scored lower on all aspects of self-management skills (as measured by the PACIC) compared to patients using primary healthcare services. (5) Participants who actively contributed to the self-help platform by posting scored higher in two empowering processes: helping others and sharing experiences. **Conclusions:** The present findings suggest that tinnitus self-management skills are independent of tinnitus duration, whereas those skills correlate negatively with illness perception. Further, clinical visits are associated with higher treatment adherence, and active self-help platform use increases feelings related to helping others and shared experiences. Taken together, these results highlight the need for fostering self-management skills and structured peer-to-peer support programs. Because this was a self-selected convenience sample of users of an online tinnitus self-help platform, the findings should be interpreted in light of this recruitment context and not generalized to the broader tinnitus population.

## 1. Introduction

Tinnitus is defined as the conscious perception of an auditory sensation without a corresponding external stimulus [1]. While the vocabulary used to describe tinnitus varies widely, it is frequently reported as a ringing, hissing, buzzing, or roaring sound within one ear, both ears, or within the head itself [2]. The etiology of tinnitus is complex and incompletely understood; however, sensorineural hearing loss, such as due to noise exposure or presbycusis, is a well-established risk factor [3]. Perhaps related to the accumulation of noise-induced damage over time, Jarach et al. [4] reported results of a meta-analysis highlighting an age-related increase in tinnitus prevalence rates, with 10% of young adults, 14% of middle-aged adults, and 24% of older adults reporting tinnitus. The severity and duration of comorbid symptoms, as well as the subsequent degree of impairment experienced, vary considerably and have proven difficult to predict at the level of any given individual [5].

Tinnitus is a highly heterogeneous condition which has resulted in the development of numerous treatment options [1,6]. Unfortunately, for many individuals experiencing tinnitus, no treatment or even combination of treatments is effective in reducing tinnitus-associated burden [7]. In these instances, finding solutions to help individuals self-manage their condition is imperative.

Actively engaging in face-to-face self-help group meetings or even online platforms is a well-accepted option for patients who do not benefit from existing interventions and must learn how to self-manage their tinnitus. Such platforms allow users to exchange knowledge and support, as well as to encourage each other to become active in their treatment journey and to better manage their health conditions [8,9,10]. For example, Nickel et al. [8] found that individuals who engage in self-help groups have a greater knowledge of their disease and treatment possibilities, while Smith et al. [9] found that self-help resources for tinnitus can empower patients and reduce the number of audiology appointments for those burdened by symptoms. In a recent study evaluating the effect of tinnitus symptom duration and self-help-seeking behavior, Probst et al. [10] reported that patients with chronic symptoms engage more in self-help, likely following a series of ineffective treatment attempts [10]. Taken together, available results suggest that self-help platforms can be effective at fostering self-management skills among individuals burdened by tinnitus.

Self-management skills have been associated with positive outcomes like greater empowerment and less obtrusive illness perception [11]. Empowerment can be operationalized as increased individual autonomy and confidence in managing health conditions [12,13], while illness perception involves being overwhelmed by the illness and its symptoms (Hagger & Orbell, 2003; Moss-Morris et al., 2002 [14,15]). Independent of tinnitus, self-management skills, empowerment, and illness perception are central to coping with chronic illnesses and improving clinical outcomes [16,17,18], as well as to overall quality of life and well-being [19,20]. Thus, it is of great relevance to explore how empowerment, self-management, and illness perception correlate with each other and how tinnitus-specific self-help platforms may foster these outcomes. Additionally, it is important to investigate whether these skills increase over time, as it may often be assumed that individuals naturally develop such competencies as they gradually learn to cope with the disease. Despite the relevance of self-management, empowerment, and illness perception, empirical studies investigating their inter-relationships in individuals with tinnitus are lacking.

Addressing this gap, the primary aim of this cross-sectional study was to investigate the association between use of an online self-help platform for people with tinnitus and self-perceptions of empowerment, symptom management skills, and illness. Specific research questions (RQ) for this study included: (RQ1) “*What is the relationship between tinnitus duration and self-management and illness perception?*”, (RQ2) “*What is the association of visiting healthcare providers to the relationship between self-management and illness perception*?”, (RQ3) “*How do the self-management skills of users of an online tinnitus self-help platform compare against the self-management skills of primary healthcare users suffering from chronic illnesses?*”, and (RQ4) “*Are empowering processes and outcomes associated with actively participating on an online self-help platform for tinnitus*?”.

Based on these questions, we formulated the following hypotheses: (H1) Longer tinnitus duration is associated with more favorable self-management and illness perception. (H2) Recent contact with healthcare providers is associated with higher scores in self-management and illness perception. (H3) Self-management scores among online platform users are comparable to or higher than those reported by primary healthcare patients with other chronic illnesses. (H4) Higher levels of active participation on the platform are positively associated with empowerment-related processes and outcomes.

## 2. Methods

### 2.1. Study Design

A cross-sectional quantitative study in the form of an online survey was conducted. The survey was designed using Qualtrics [21] and distributed via the online platform Tinnitus Talk on 8 May 2023. Tinnitus Talk is an online forum where members can discuss recent scientific findings, share their experiences of living with tinnitus and coping strategies, and provide mutual support. Participants of this study included people who reported to suffer from tinnitus, who clicked on a link to the study materials within a thread related to research news. Participants were recruited through an online tinnitus self-help platform. This constitutes a convenience sample of platform users rather than a population-based tinnitus sample. The study link was accessible for 19 weeks, from 8 May to 18 September 2023. Non-personally identifiable user information (e.g., no IP or home addresses, date of birth, contact information, names) was collected and later stored on a secure server provided by the University of Twente.

To determine the required sample size for detecting statistically significant correlations, an a priori power analysis was conducted using G*Power 3.1 (Faul et al., 2009 [22]). The analysis was based on an exact test family with a two-tailed distribution, targeting the detection of a small-to-moderate correlation (Pearson’s r = 0.3). A significance level (α) of 0.05 and a desired statistical power (1 − β) of 0.95 were specified. A power level of 0.95 was selected to minimize the probability of a Type II error (β = 0.05). Although 80% power (β = 0.2) is commonly used, methodological critiques have highlighted the shortcomings of underpowered studies, particularly their reduced likelihood of detecting real effects and their contribution to non-reproducible results [23]. Therefore, setting β at 0.05 was considered a more conservative approach to reduce the chance of false negatives. The results indicated that a minimum of 138 participants would be required to reliably detect a correlation of this magnitude under the given parameters. The a priori power analysis was conducted for the primary correlational objective of the study (RQ1); the remaining analyses should therefore be interpreted as secondary/exploratory.

Informed consent, which explained the study’s purpose, the risks and benefits of participation, and the participants’ rights, was obtained before data collection. Ethics approval was obtained on 23 March 2023 by the Ethics Committee from the Faculty of Behavioral, Management and Social Sciences of the University of Twente, project number 230236. This study followed the STROBE (Strengthening the Reporting of Observational Studies in Epidemiology) guidelines for cross-sectional studies to ensure transparent and comprehensive reporting [24], available in Supplemental Table S1.

### 2.2. Procedure and Instruments

After agreeing to participate in the study, participants were invited to complete a survey battery consisting of five questionnaires, as well as a set of questions related to the participants’ activity on Tinnitus Talk. The survey could only be completed if all questions were answered. However, for each question, an answering option “(I do not know/Prefer not to say/Other)” was given, which is shown in Table 1 as (missing). The motivation for each questionnaire, in addition to a detailed description, is provided below.

#### 2.2.1. TSCHQ

The Tinnitus Sample Case History Questionnaire (TSCHQ) was used to collect both demographic information and information about the participant’s tinnitus history [25] and consists of 35 items in the form of both open-ended and multiple-choice questions. The TSCHQ collects general information like age, gender and handedness, as well as tinnitus-specific information like type of perceived noise (e.g., white noise, pure tone), pitch frequency and loudness. General information about participants’ demographics from the TSCHQ is shown in Table 1. The TSCHQ was adapted in this study to minimize the time required for a participant to complete the survey, while collecting all the information relevant for this study. Specifically, four original items of the TSCHQ that were deemed irrelevant to this study’s goals were excluded, while four others were added by the researchers. Changes from the TSCHQ are described below.

The original item 13 “Please describe in your own words what your tinnitus usually sounds like:” and item 25 “Does medication have an effect on your tinnitus?” were excluded because analyzing textual data was beyond the scope of this project. Furthermore, item 16 “What per cent of your total awake time, over the last month, have you been aware of your tinnitus?” and item 17 “What percent of your total awake time, over the last month, have you been annoyed, distressed, or irritated of your tinnitus?” were excluded to prevent duplication.

The questions: “In which country do you currently reside?”, “Which statement best describes your current employment status?” and “What is the highest education level you have achieved?” were added to get a more detailed picture of the participant’s demographics. Finally, the question “Do you currently receive any form of treatment for your tinnitus?” was added to compare participants who received tinnitus-specific treatment (RQ2).

#### 2.2.2. Platform Activity

This component consisted of three custom-designed questions created specifically for this study to assess participants’ engagement with the Tinnitus Talk platform (see Supplemental Figure S1). First, participants were asked, “How long have you been participating on this platform?”, where they could indicate the number of months and years since joining (or select “I do not know”). Second, platform usage frequency was assessed with the question, “How often do you visit Tinnitus Talk?”, with response options ranging from “I do not use it” to “More than once a day” on a 7-point scale. Third, posting behavior was evaluated with two linked prompts, “Do you contribute to the platform by posting?” and “How often (on average) do you contribute to the platform?”, with frequency options from “Less than once a month” to “More than once a day.” Responses to the posting-related questions were used to differentiate between active and passive users for the purposes of RQ4. Because these items as well as the TSCHQ were designed for descriptive profiling rather than as a unidimensional scale, and were specific to this platform, no internal consistency measure was calculated.

#### 2.2.3. Brief IPQ

The Brief Illness Perception Questionnaire [26] was administered to assess participants’ cognitive and emotional representations of their tinnitus. The IPQ questions are formulated with the word “illness”, but this was replaced with the word “tinnitus” as suggested by Broadbent et al. [26]. For example, the first question, “How much does your illness affect your life?” was reformulated to “How much does your tinnitus affect your life?”. In its original form, the Brief IPQ is a nine-item scale where answers are given on a scale from 0 to 10, except for the last item where three open-answer possibilities are given [26]. For this survey, the last item “Please list in rank-order the three most important factors that you believe caused your illness. The most important causes for me:” was removed because analyzing textual data was beyond the scope of this project, and we aimed to reduce the time required for participants to complete the survey used in this study. The questionnaire showed moderate reliability in this sample, with Cronbach’s alpha of 0.69.

#### 2.2.4. PACIC

The Patient Assessment of Chronic Illness Care (PACIC) is a 20-item scale measuring patients’ assessment of patient-centered and proactive care for chronically ill people [27]. The 20 items were answered on a Likert scale ranging from 1 (“Almost never”) to 5 (“Almost always”). Examples of PACIC questions include: “I was asked to talk about my goals in caring for my tinnitus” or “I was encouraged to attend programs in the community that could help me”. Moreover, the items are distributed over 5 subscales assessing “patient activation”, “design/decision support”, “goal setting”, “problem-solving/contextual counseling”, and “follow-up/coordination” (Glasgow et al., 2005 [27]). As the PACIC focuses on healthcare encounters over the last six months, the filter question, “Did you visit any healthcare provider (e.g., GP, ear–nose–throat doctor, neurologist, psychiatrist, audiologist, dentist, etc.) specifically for your tinnitus?”, with four response options, was added: (1) No, never visited a healthcare provider, (2) Yes, in the last 6 months, (3) Yes, between 6 and 12 months ago and (4) Yes, over 12 months ago (see Table 1). These response options were added to increase the response rate, as tinnitus often results in patients having not visited a healthcare provider specifically for the condition for a long time. The questionnaire was not presented to participants who reported not having yet visited a healthcare provider for their tinnitus. For this sample, Cronbach’s α of the PACIC was 0.88.

#### 2.2.5. PIH Scale

The Partners in Health (PIH) Questionnaire evaluated participants’ ability to self-manage their chronic conditions and assessed their illness-related knowledge [28]. The questionnaire consists of 12 items, with response options ranging from 0 to 8, with categories depending on the items. For the first two items, the categories range from “0—Very little” to “8—A lot”, for item 3 to item 8 the categories are “0—Never” to “8—Always”, and for items 9 to 12 the categories range from “0—Not very well” to “8—Very well”. In the present study, this questionnaire was used to evaluate self-management ability, which was in turn used to further describe the level of empowerment in the participants. A key difference between the PACIC and the PIH is that the PACIC assesses the quality and alignment of chronic illness care with the Chronic Care Model from the patient’s perspective, while the PIH evaluates patients’ knowledge, self-management behaviors, and the impact of their chronic condition on their lives. For this sample, Cronbach’s α of the PIH was 0.78.

#### 2.2.6. Empowerment Questionnaire

This questionnaire, developed by van Uden-Kraan et al. [29], measured the empowerment processes and outcomes associated with platform engagement. The questionnaire was developed to evaluate these constructs among users of online self-help platforms suffering from chronic conditions like breast cancer, arthritis, and fibromyalgia [29]. Empowerment is understood both as a process where individuals or groups take control over their lives and manage disease, and as an outcome representing a state of psychological enablement [30]. The empowering processes scale consists of 29 items distributed over five subscales: “exchange information”, “social support”, “other support”, “helping others”, and “shared experiences”. The items were measured on a 4-point scale ranging from “seldom to never” to “often”. The empowering outcomes scale consists of 38 items distributed over seven subscales: “feeling better informed”, “confidence in physician”, “illness acceptance”, “confidence in treatment”, “optimism”, “self-esteem”, and “well-being”. Items from this scale were measured on a 5-point scale ranging from “completely disagree” to “completely agree”. Cronbach’s α for the empowering processes and outcomes were 0.93 and 0.94, respectively.

### 2.3. Data Analysis

#### Statistical Methods

Numeric data were analyzed descriptively using means and standard deviations (SD). For categorical data, counts were determined for each category, followed by the corresponding percentages. Only complete cases, where participants filled out the entire questionnaire, allowing us to calculate sum scores, were included in each analysis. The online survey required a response to each item before participants could proceed. However, some items allowed responses such as “I do not know,” “Prefer not to say,” or “Other.” For descriptive and inferential analyses, these responses were treated as not available for the relevant variable and are indicated as N/A in the tables. Analyses were therefore conducted using complete data for the variables required in each specific analysis, resulting in varying denominators across research questions. For RQ1, the correlation between the PIH, IPQ, and PACIC subscales of tinnitus duration was calculated with Spearman’s method. P-values were adjusted with the Hochberg method to account for multiplicity bias [31]. The visualization of correlations was conducted with the corrplot package [32].

To complement the zero-order correlation analysis, we also estimated a partial-correlation network. Whereas pairwise correlations quantify marginal associations, the partial-correlation network isolates the association between each pair of variables after adjusting for all others, which helps to distinguish direct from indirect relationships in this multivariate set. The network was estimated with the “ggModSelect” algorithm, a Gaussian graphical stepwise model-selection approach implemented in the qgraph package (Epskamp et al., 2012 [33]). The network is descriptive and hypothesis-generating rather than confirmatory. Stability of network parameters and of centrality estimates was assessed with non-parametric bootstrapping (*n* = 1000) using the bootnet package (Epskamp et al., 2017 [34]); centrality indices, edge weights, and bootstrap estimates are shown in Supplemental Figures S3 and S4.

For RQ2, Analysis of Variance (ANOVA), followed by Tukey’s post hoc analysis, was conducted to investigate whether participants who reported visiting a healthcare provider in the past scored differently across the IPQ and the different subscales of the PIH. For RQ3, we used a one-sample *t*-test to compare our sample means and SDs of the PACIC subscales with the population means and SDs reported by Glasgow et al. [27] for the same subscales. For RQ4, we conducted a non-paired *t*-test to compare differences in empowering processes and outcomes between active and passive users of the Tinnitus Talk. Data analysis was done using R (version 4.2.0) and RStudio (Version 2023.03.1+446).

## 3. Results

The Results Section is organized into five subsections. The first presents participants’ sociodemographic characteristics and the distributions of scores on the PIH, PACIC, IPQ (including its subscales), and the Empowering Processes and Outcomes Scale. The remaining four subsections correspond to the four research questions (RQs) outlined in the introduction. Because analyses were conducted using complete data for the variables required in each specific analysis, analytic sample sizes differed somewhat across research questions and subscales.

### 3.1. Participant Characteristics

**Figure 1:**
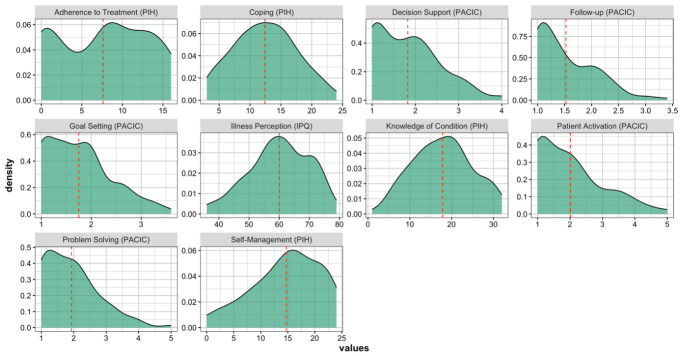
Density plots of the Illness Perception Questionnaire (IPQ), Partners in Health (PIH), and Patient Assessment of Chronic Illness Care (PACIC). Note: Red dashed lines indicate the mean.

**Figure 2:**
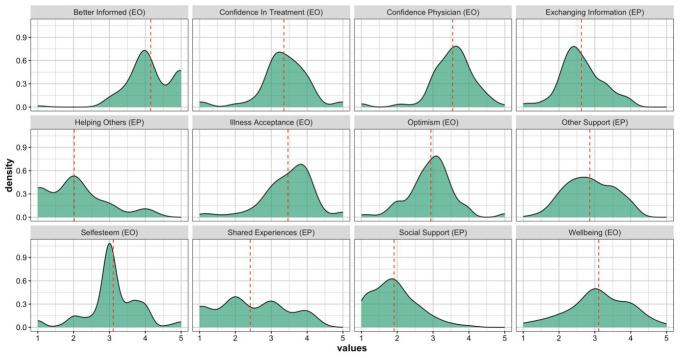
Density plots of the empowering processes (EP) and outcomes (EO). Note: Red dashed lines indicate the mean.

This study included 152 participants, with most participants being men (75%), active contributors to the Tinnitus Talk (43%), and from Anglo-Saxon-speaking countries (see Supplemental Figure S1). Table 1 summarizes the most relevant sociodemographic information of the sample. Figure 1 shows the distribution of the IPQ and the subscales of the PIH and PACIC. Figure 2 shows the distribution of the empowering processes and outcomes.

### 3.2. RQ1: The Correlation Between Self-Management, Illness Perception, and Tinnitus Duration

**Figure 3:**
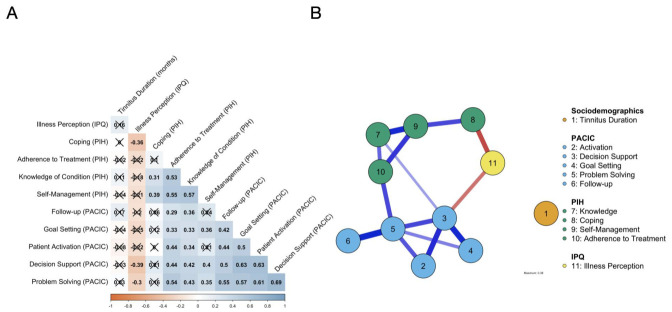
Correlation between PIH, IPQ, PACIC, and tinnitus duration. Note: Panel (**A**) shows the univariate correlation between variables. Red colors indicate negative correlations and blue colors indicate positive correlations. Crossed cells indicate non-statistically significant correlations after adjusting *p*-values with the Hochberg method. Panel (**B**) shows the partial correlation between variables, that is, the correlation between two variables after accounting for covariates. Red lines indicate negative partial correlations and blue lines indicate positive partial correlations. PACIC = Patient Assessment of Chronic Illness Care, PIH = Partners in Health, IPQ = Illness Perception Questionnaire. Tinnitus duration represented the number of months since the onset of tinnitus.

First, we conducted univariate correlations between the IPQ, the subscales of the PIH and the PACIC, and self-reported tinnitus duration (in months) to better understand whether chronicity is associated with better self-management skills. Analyses were based on participants with complete data for each variable pair; pairwise n varied across correlations and was maximal at *n* = 152. A total of 55 pairwise Spearman correlations were conducted, with 31 (56.4%) being statistically significant after adjusting for multiple comparisons (see Figure 3A). Most statistically significant correlations were observed between self-management items from the PIH and PACIC, with correlation coefficients ranging from 0.31 and 0.69. No significant correlation between tinnitus duration and any of the scales was observed, and the IPQ was negatively correlated with decision support, problem-solving (PACIC), and coping skills (PIH).

Next, we performed partial correlations with the same variables to identify the correlation between two variables after controlling for the remaining covariates. The results are presented in Figure 3B, with different nodes representing the different scales or the duration of tinnitus, and the links between nodes representing the correlations between them. In total, 14 partial correlations were observed, with 12 positive and 2 negative. The positive correlations were mostly between self-management skills between the PIH and PACIC, and the negative correlations were between the IPQ, decision support (PACIC), and coping skills (PIH), corroborating the previous analysis. No significant correlations were observed between tinnitus duration and the subscales. The subscale “follow-up” from the PACIC showed the highest centrality.

### 3.3. RQ2: The Association Between Self-Management, Illness Perception and Visiting Healthcare Providers

**Figure 4:**
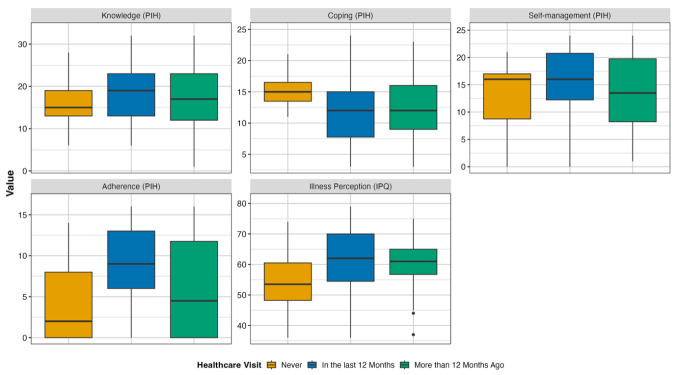
Boxplots showing the distribution of the PIH and of the IPQ by healthcare visit. Note: The y-axis scale varies for each panel reflecting the range of scores for each (sub)scale, so the values on the y-axis may differ between panels. Participants completed the PACIC based on their impressions from their last healthcare visit specifically for tinnitus. Those who reported never having visited a healthcare provider for tinnitus (see Table 1) did not complete this questionnaire and thus were excluded from this analysis. PIH = Partners in Health, IPQ = Illness Perception.

Next, we compared self-management skills as assessed using the PIH and tinnitus perception as assessed using the IPQ among participants who reported having visited healthcare practitioners for their tinnitus compared to participants who did not. The PACIC was excluded from this analysis because the questionnaire was not delivered to participants who reported not having received specific healthcare for their tinnitus (see Section 2). A total of 15 participants reported having never received tinnitus-specific healthcare, while 80 reported having received care in the last 12 months, and 38 reported having received care more than 12 months ago. Figure 4 shows the scores per group. We conducted ANOVAs to compare group means (see Table 2) and post hoc comparisons (see Table 3). After adjusting for multiple comparisons, we found that the PIH was higher in the group that visited a healthcare provider in the last 12 months compared to those who did not (mean difference = 4.91, *p* = 0.003), and was significantly lower in the group that visited a healthcare provider more than 12 months ago compared to those who did in the last 12 months (mean difference = −3.23, *p* = 0.009). Because the “Never visited” group included only 15 participants, estimates involving this group should be interpreted with caution given the limited precision and wide confidence intervals.

### 3.4. RQ3: Comparing Self-Management Skills of Users of an Online Tinnitus Self-Help Platform Against Primary Healthcare Users

**Figure 5:**
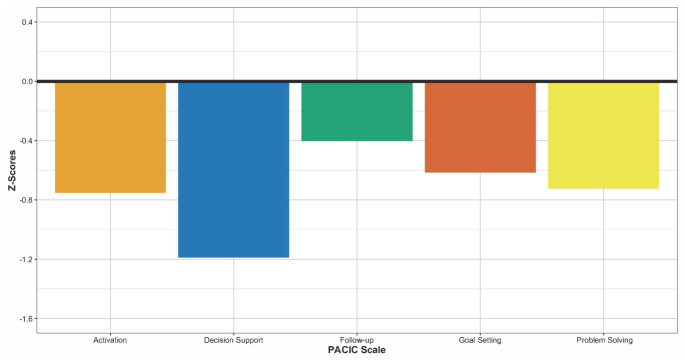
Bar chart with z-scores for the five PACIC subcomponents comparing individuals suffering from tinnitus and primary healthcare users. Note: The figure shows the z-score compared with normative values reported by Glasgow et al. [27]. All the subcomponents have negative z-scores, indicating that their means are below the expected population means (µ). The length of each bar corresponds to the magnitude of deviation from the mean, with longer bars indicating a greater difference.

We used one-sample *t*-tests to compare the mean and standard deviations with the scores reported by Glasgow and colleagues (2005) [27] of the PACIC. This study reported the development and validation of the PACIC among primary healthcare users in the United States suffering from chronic diseases such as hypertension, arthritis, depression, diabetes, asthma, and pain (Glasgow et al., 2005 [27]). We found that online users of the self-help platform for tinnitus scored lower in all five dimensions of the PACIC, with the starkest difference for decision support (see Table 4 and Figure 5). Given the differences in country, healthcare system, disease, and recruitment setting between our sample and the Glasgow et al. (2005) [27] reference population, this comparison should be interpreted as hypothesis-generating; see the Discussion for a detailed caveat. Cohen’s d ranged from −1.16 (activation) to −5.32 (follow-up).

### 3.5. RQ4: Empowering Processes and Outcomes Associated with Actively Participating on an Online Self-Help Platform for Tinnitus

We compared five empowering processes and seven empowering outcomes between active and passive users of Tinnitus Talk. The results from 122 participants were included in this analysis (see Table 5). The analytic sample comprised 53 active and 69 passive users. After correcting for multiple comparisons, we found that “helping others” and “sharing experiences” empowering processes showed statistically significant differences, with active users scoring higher in both domains. The magnitude of these effects was large for helping others (Cohen’s d ≈ 0.80) and medium-to-large for shared experiences (d ≈ 0.66). The data suggested no differences between active and passive users for any empowering outcomes.

## 4. Discussion

In this cross-sectional study, we investigated the association between empowering processes and outcomes, self-management skills, and illness perception among users of an online self-help platform for tinnitus. We identified several key findings: (1) contrary to expectations, tinnitus duration was not associated with better self-managing skills (H1, see Figure 3), (2) when comparing participants who reported visiting a healthcare provider for their tinnitus with participants who did not, we observed that adherence to treatment was scored higher among participants with clinical visits (H2, see Figure 4, Table 2 and Table 3), (3) tinnitus representations, as assessed using the IPQ and operationalized as the participants’ cognitive and emotional perception of their tinnitus, were negatively associated with self-managing conditions (H1, see Figure 3), (4) contrary to our hypothesis, participants of this study scored lower in all dimensions of self-management skills (according to the PACIC) when compared to patients using the primary healthcare system (H3, see Table 4 and Figure 5), and (5) participants who reported actively contributing to the self-help platform with posts scored higher in two empowering processes, helping others and shared experiences (H4, see Table 5). The implications of these findings are discussed below.

The lack of evidence in the present study for an association between self-management skills and tinnitus duration is a key finding with clinically meaningful implications. Whereas tinnitus habituation, defined as a decrease in responsiveness to the phantom perception [35], is an expected outcome of living with tinnitus for most individuals, our results suggest that the same cannot be said about self-managing skills. Self-management skills, such as knowledge of one’s disease, confidence, and willingness to manage one’s health, goal setting with and without the assistance of healthcare providers, and problem-solving abilities, are central to quality of life and well-being for individuals suffering from chronic diseases [19]. Thus, developing and enhancing self-managing skills in individuals burdened by tinnitus should be encouraged regardless of the disease duration. Within this sample, self-management scores were lower than those reported for primary care users with chronic conditions (see Figure 5), which should be interpreted with the caveats described in the RQ3 discussion below. Although some programs and interventions exist, there remains a need for more targeted efforts to promote self-management in chronic tinnitus patients.

Self-managing skills are vital under the Chronic Care Model (CCM), as they promote patient-centered care and long-term management of chronic diseases. The CCM framework, supported by programs like the Stanford Model and Community-based Transition Model, aims to reorient healthcare from acute to long-term care in response to increased life expectancy and the prevalence of chronic conditions [36]. This approach has been proposed in the context of audiological practice to address specific implementation challenges [37]. For example, patient-centered, self-management support has been an integral part of aural rehabilitation and communication programs for a long time [37]. These programs, frequently conducted in group settings and encompassing a variety of content related to patient activation, typically provide education on hearing loss and hearing aid use, offer communication strategies, teach speechreading techniques, and incorporate relaxation methods, mindfulness practices, and psychosocial support. However, similar structured programs oriented towards chronic care are notably lacking for individuals with tinnitus, highlighting a significant gap in the availability of comprehensive care for this condition.

Developing self-efficacy, continuous education, and active health management, self-help platforms may enhance resilience and self-management, aligning with CCM goals [12]. Despite the recognition of CCM’s relevance for managing conditions such as diabetes, hypertension, and cardiovascular diseases [38], empirical evidence in audiological services, particularly for tinnitus, remains scarce. This study is the first to use the PACIC, an instrument developed to evaluate the implementation of the CCM, to evaluate self-managing skills in tinnitus patients, highlighting the need for further studies to assess the effectiveness of these models in clinical practice.

This comparison should be interpreted as hypothesis-generating rather than as evidence that tinnitus patients receive poorer chronic care support. The PACIC was developed and benchmarked in U.S. primary care populations with chronic diseases such as hypertension, diabetes, arthritis, asthma, depression, and pain (Glasgow et al., 2005 [27]), and several factors beyond the condition itself may contribute to the observed differences, including country- and healthcare-system differences, disease-specific care pathways, the contrast between online and clinic-based recruitment, the period over which care was assessed, and the fact that in our sample the PACIC was completed only by participants who had visited a tinnitus-specific healthcare provider. Differences observed here should therefore motivate targeted evaluation of chronic care implementation for tinnitus rather than be taken as direct evidence of inferior care.

We also found that cognitive and emotional representations of tinnitus, as measured by the brief IPQ, were negatively associated with self-management skills, like decision support, coping, problem-solving skills (see Figure 3A) and goal setting (Figure 3B). Aligned with our results, Vollmann et al. [39] showed that negative illness representations, especially the attribution of severe consequences and strong emotional responses, are associated with maladaptive coping strategies and poorer tinnitus adjustment. Similarly, ref. [40] demonstrated that these representations of serious consequences, also measured by the IPQ, are significant predictors of increased psychological morbidity, accounting for a substantial portion of the variance in both anxiety and depression among tinnitus patients. Lastly, ref. [41] found empirical support for a model of tinnitus distress, emphasizing the significant role of negative representations of tinnitus in maintaining tinnitus distress. The study confirms that these negative representations are closely interrelated with other psychological factors, such as attention and negative thinking, and play a central role. Overall, there seems to be mounting evidence that the cognitive and emotional representations of tinnitus play a central role in the associated burden, and our study suggests that such representations are also negatively associated with self-management skills.

In addition to the association between platform usage and self-managing skills, our results also suggest an association between visiting a healthcare provider and higher treatment adherence (see Table 3). Even though no single treatment has proven effective in reducing tinnitus symptoms in a majority of people, several treatment methodologies, such as medication or cognitive behavior therapy for potentially accompanying mood disorders, adopting sleep hygiene techniques to improve sleep quality, or hearing aids to account for hearing loss [42], have been shown to reduce tinnitus-related burden. Furthermore, current clinical guidelines recommend addressing the often co-occurring comorbidities, like health-related anxiety and sleep-related problems, to improve patients’ quality of life and well-being [43]. Although beyond the scope of this project, we speculate that the observed increased adherence to treatment among patients visiting healthcare providers may be indicative of individuals’ willingness to identify and adhere to treatment strategies to manage comorbidities. Given the cross-sectional design, the direction of this association cannot be established: recent clinical contact may support adherence, but more adherent individuals may also be more likely to maintain clinical follow-up. In addition, the comparison involves a small “Never visited” group (*n* = 15), so these estimates should be interpreted with caution.

Regarding empowering processes and outcomes, our results suggest that both active and passive users benefit from interactions on the platform similarly, except for two notable exceptions. Namely, we observed that active users scored higher in the subscales of helping others and shared experiences. These results are aligned with those reported by van Uden-Kraan et al. [29], where increased empowerment was found among active contributors to self-help platforms for breast cancer survivors, fibromyalgia, and arthritis. Given the lack of satisfactory and equitable access to healthcare [44,45], the limited number of specialized healthcare providers to support individuals burdened by tinnitus [46], and the emergence of programs leveraging communal aspects, capitalizing on (online) community-building and peer-to-peer support emerges as an alternative path for self-management and chronic care.

For example, the EMPOWER program [47] builds on clinical science demonstrating the effectiveness of brief psychosocial interventions delivered by a non-specialist, frontline-care workforce. This program is aimed at training motivated non-specialists to provide necessary support to individuals suffering from mental health problems, thereby increasing the accessibility of treatment while minimizing the healthcare burden. These non-specialists are trained with syllabus materials created by clinical experts and further trained by clinical psychologists on delivering psychosocial interventions. EMPOWER has been tested in different cohorts suffering from various mood disorders, but similar programs specifically for individuals burdened by tinnitus have not been explored. Based on the positive results of active engagement in the Tinnitus Talk platform and the empowering processes identified in this study, we recommend future research to develop and validate programs through which members of online tinnitus communities are trained to provide peer-to-peer support to those struggling to cope with the condition.

## 5. Limitations and Generalizability

It is important to consider the characteristics of our sample (see Table 1) and the generalizability of our findings when interpreting these results. Recruitment through an online tinnitus self-help platform may have introduced selection bias, as individuals active in such communities may differ from the broader tinnitus population in symptom severity, coping style, help-seeking behavior, health literacy, and willingness to discuss tinnitus-related experiences. Previous work has suggested that the user demographics of platforms such as Tinnitus Talk can differ significantly from those of clinical patient populations (Probst et al., 2017 [10]), although other studies have found that online samples may resemble clinical samples reasonably well in some respects (Russell et al., 2023 [48]). The direction of this potential bias remains unclear: platform users may score higher in self-management because they have access to specialized information and peer exchange, but they may also be overrepresented by individuals who seek out such platforms precisely because they experience greater difficulty with self-management or habituation. In addition, the online survey format may have underrepresented individuals with lower digital access or proficiency. Although we cannot fully exclude the possibility that some participants took part without having tinnitus, this seems unlikely given that recruitment took place through a specialized tinnitus platform and the survey included tinnitus-specific questions. All measures were based on self-report and are therefore subject to biases such as recall inaccuracies, social desirability, and subjective interpretation. Finally, because of the cross-sectional design, no causal conclusions can be drawn from the observed associations. Longitudinal, interventional, and naturalistic designs may help clarify the mechanisms underlying self-management skills and disease burden (Grosz et al., 2024 [49]). Formal assessments of normality and homoscedasticity were not performed for the ANOVAs and *t*-tests. At the present sample sizes, parametric tests of this family are known to be relatively robust to moderate departures from normality and to unequal variances when group sizes are comparable (Lumley et al. 2002 [50]), so we do not expect these assumption violations to materially affect the main inferences. The results should nonetheless be interpreted with caution, particularly for comparisons involving the smallest subgroups (e.g., the “never visited” healthcare provider group, *n* = 15), where both power and robustness are limited.

## 6. Future Directions

Future research should aim to replicate these findings in larger and more representative samples, including individuals recruited from clinical settings, who often experience more severe and persistent forms of tinnitus. Expanding the sample beyond online platforms will improve generalizability and may help identify specific groups with heightened need for support in self-management and empowerment. Longitudinal and mixed-method designs could provide a clearer understanding of the causal pathways between online engagement, empowerment processes, and health-related outcomes. The consistently low PACIC scores observed suggest a need to develop and evaluate interventions aimed at improving shared decision-making and patient-centered care. These interventions could target both healthcare providers and patients, focusing on communication strategies, goal setting, and coordinated care planning. Notably, the association between active engagement on Tinnitus Talk and higher levels of empowering processes indicates a promising opportunity to leverage peer-driven support models. Public health initiatives could identify and support highly engaged users as peer leaders or facilitators within structured programs, such as EMPOWER or similar models, to promote qualified, experience-based guidance. Both provider-oriented and peer-led interventions could be designed and tested using established frameworks [51]. These future efforts can help translate (online) community engagement into tangible, scalable improvements in self-management and care for people living with tinnitus.

## 7. Conclusions

The purpose of this study was to investigate the association between empowering processes and outcomes, self-management, and illness perception among users of an online self-help platform for tinnitus. Briefly, our results suggest that tinnitus representations are negatively associated with self-management skills, that longer tinnitus duration does not correspond with better self-management, and that illness perception is similarly linked to lower self-management abilities. Additionally, clinical visits were associated with higher treatment adherence, and active platform users reported higher scores in two empowering processes, helping others and shared experiences. While these data do not establish how self-management skills develop over time, they suggest that self-management may benefit from active support rather than emerging spontaneously with disease duration. Consequently, promoting self-management practices may be relevant regardless of tinnitus duration. Future directions include testing structured peer-to-peer support programs, as training motivated non-specialists could provide necessary support while reducing healthcare burdens. Longitudinal and interventional studies are also needed to understand the mechanisms behind improved self-management skills and reduced tinnitus-related burden.

## Figures and Tables

**Table 1:** Demographics.

Characteristic	N = 152 ^1^
**Gender**	
Female	33 (22%)
Male	113 (75%)
Non-Binary	4 (2.7%)
N/A	2
**Age**	40 (13)
N/A	9
**Employment Status**	
Not Working (Disabled)	13 (9.1%)
Not Working (Looking for Work)	7 (4.9%)
Not Working (Other)	10 (7.0%)
Not Working (Retired)	11 (7.7%)
Not Working (Temporary Layoff from a Job)	1 (0.7%)
Prefer Not to Answer	7 (4.9%)
Working (Paid Employee)	82 (57%)
Working (Self-Employed)	12 (8.4%)
(N/A)	9
**Education Level**	
Lower Secondary (Middle School)	3 (2.1%)
University (Higher Degree)	103 (73%)
Upper Secondary (High School)	35 (25%)
(N/A)	11
**Visting Frequency**	
I do not use it	2 (1.7%)
Less than once a month	5 (4.1%)
Once a month	9 (7.4%)
Once a week	19 (16%)
More than once a week	34 (28%)
Once a day	14 (12%)
More than once a day	38 (31%)
(N/A)	31
**Active Contribution to Tinnitus Talk**	
No, I never posted on Tinnitus Talk	34 (28%)
Yes, but not anymore	35 (29%)
Yes, I am an active poster on Tinnitus Talk	53 (43%)
(N/A)	30
**Posting Frequency on Tinnitus Talk**	
Less than once a month	70 (58%)
Once a month	19 (16%)
Once a week	21 (17%)
Once a day	5 (4.1%)
More than once a day	6 (5.0%)
(N/A)	31
**Previous Visit to a Healthcare Provider Specifically for Tinnitus**	
No, never visited a healthcare provider	15 (11%)
Yes, between 6 and 12 months	10 (7.5%)
Yes, in the last 6 months	70 (53%)
Yes, over 12 months ago	38 (29%)
(N/A)	19

^1^ n (%); mean (SD). IPQ = Illness Perception Questionnaire, PIH = Partners in Health, PACIC = Patient Assessment of Chronic Illness Care; N/A = not available for analysis (e.g., responses such as “I do not know”, “Prefer not to say”, or “Other”).

**Table 2:** ANOVA comparing self-management skills and tinnitus perception among patients who visited a healthcare provider.

In the Last 12 Months N = (80)	More than 12 Months Ago N = (38)	Never N = (15)	F
Mean (SD)	Mean (SD)	Mean (SD)	
**Knowledge (PIH)**			
18.58 (7.01)	17.21 (7.54)	13.43 (6.58)	F(2, 112) = 1.09
**Coping (PIH)**			
11.58 (5.23)	12.80 (4.91)	15.27 (2.69)	F(2, 113) = 3.64 *
**Self-Management (PIH)**			
15.60 (5.84)	13.44 (6.84)	13.43 (6.58)	F(2, 113) = 1.70
**Adherence (PIH)**			
9.17 (4.89)	5.94 (5.80)	4.27 (4.76)	F(2, 115) = 8.11 ***
**Illness Perception (IPQ)**			
60.83 (10.26)	60.44 (8.73)	54.57 (9.87)	F(2, 124) = 2.46

Note: PIH = Partners in Health, IPQ = Illness Perception Questionnaire, SD = Standard Deviation. F statistics are reported followed by between- and within-group degrees of freedom. * indicates a *p*-value < 0.05, *** indicates a *p*-value < 0.001.

**Table 3:** Tukey HSD post hoc comparisons of self-management skills and tinnitus perception among patients who visited a healthcare provider.

Comparison	Estimate	95% CI	*p*-Value	q-Value ^1^
**Coping (PIH)**				
In the Last 12 Months—Never	−3.69	[−7.01, −0.37]	0.025	0.1
More than 12 Months Ago—Never	−2.47	[−6.05, 1.12]	0.2	0.5
More than 12 Months Ago—In the Last 12 Months	1.22	[−1.20, 3.65]	0.5	0.5
**Adherence (PIH)**				
In the Last 12 Months—Never	4.91	[1.42, 8.39]	0.003	0.010 *
More than 12 Months Ago—Never	1.67	[−2.12, 5.47]	0.5	0.5
More than 12 Months Ago—In the Last 12 Months	−3.23	[−5.80, −0.67]	0.009	0.019 *

Note: PIH = Partners in Health, ^1^ Hochberg correction for multiple testing. * indicates a *p*-value < 0.05.

**Table 4:** Comparison of the PACIC subscales from the sample means with the population mean of primary healthcare users in the United States reported by Glasgow and colleagues (2005) [27].

	Mean	µ	Difference	95% CI (Difference)	t(df)	q-Value	Cohen’s d
Activation	2.01	2.6	−0.59	[1.8, 2.2]	−6.16 (113)	<0.001 ***	−1.16
Decision Support	1.82	2.99	−1.17	[1.7, 1.9]	−16.50 (110)	<0.001 ***	−3.15
Goal Setting	1.75	3.13	−1.38	[1.6, 1.9]	−22.10 (108)	<0.001 ***	−4.25
Follow-Up	1.53	2.97	−1.44	[1.4, 1.6]	−26.98 (103)	<0.001 ***	−5.32
Problem-Solving	1.93	2.43	−0.50	[1.8, 2.1]	−6.08 (102)	<0.001 ***	−1.20

Note: µ = population mean, CI = confidence interval, t(df) = t-value with degrees of freedom in parentheses, q-values were obtained from a one-sample *t*-test after correction for multiple testing with the Hochberg method. *** indicates a *p*-value < 0.001.

**Table 5:** Comparison of active and passive web forum users.

Characteristic	Active User, N = 53 ^1^	95% CI	Passive User, N = 69 ^1^	95% CI	*p*-Value ^2^	q-Value ^3^
**Exchange Information (EP)**	2.56 (0.63)	[2.4, 2.7]	2.68 (0.58)	[2.5, 2.8]	0.3	>0.9
(Missing)	5		16			
**Social Support (EP)**	2.03 (0.58)	[1.9, 2.2]	1.84 (0.70)	[1.6, 2.0]	0.2	>0.9
(Missing)	3		21			
**Other Support (EP)**	2.89 (0.63)	[2.7, 3.1]	2.81 (0.69)	[2.6, 3.0]	0.6	>0.9
(Missing)	2		16			
**Helping Others (EP)**	2.34 (0.77)	[2.1, 2.6]	1.72 (0.78)	[1.5, 1.9]	<0.001	0.001 *
(Missing)	3		17			
**Shared Experiences (EP)**	2.73 (0.83)	[2.5, 3.0]	2.11 (1.01)	[1.8, 2.4]	0.001	0.015 *
(Missing)	3		22			
**Better Informed (EO)**	4.10 (0.72)	[3.9, 4.3]	4.22 (0.63)	[4.0, 4.4]	0.4	>0.9
(Missing)	2		16			
**Confidence in Physician (EO)**	3.55 (0.63)	[3.4, 3.7]	3.54 (0.65)	[3.4, 3.7]	>0.9	>0.9
(Missing)	3		20			
**Illness Acceptance (EO)**	3.41 (0.75)	[3.2, 3.6]	3.50 (0.73)	[3.3, 3.7]	0.5	>0.9
(Missing)	3		18			
**Confidence in Treatment (EO)**	3.38 (0.72)	[3.2, 3.6]	3.32 (0.73)	[3.1, 3.5]	0.7	>0.9
(Missing)	2		19			
**Optimism (EO)**	2.98 (0.60)	[2.8, 3.2]	2.90 (0.68)	[2.7, 3.1]	0.5	>0.9
(Missing)	3		17			
**Self-esteem (EO)**	3.15 (0.70)	[3.0, 3.4]	3.06 (0.79)	[2.8, 3.3]	0.5	>0.9
(Missing)	3		17			
**Well-being (EO)**	3.28 (0.92)	[3.0, 3.5]	2.95 (0.82)	[2.7, 3.2]	0.060	0.6
(Missing)	3		17			

Note: ^1^ Mean (SD), ^2^ 2-sample *t*-test, ^3^ q-values were obtained with the Hochberg correction for multiple testing. CI = confidence interval, EP = empowering processes, EO = empowering outcomes. * indicates a *p*-value < 0.05.

## Data Availability

The raw data supporting the conclusions of this article will be made available by the authors on request.

## Supplementary Materials

The following supporting information can be downloaded at: https://www.mdpi.com/article/10.3390/jcm15114043/s1, Figure S1: Average Time to Complete the Survey; Figure S2: Country of Residence of Participants; Figure S3: Centrality Indices of Network Nodes from Figure 3B; Figure S4: Edge Weights and Bootstrap Estimates for Network Stability; Table S1: STROBE Checklist.
