# Supplementary material for: Empowerment, Self-Management and Illness Perception of Users of an Online Self-Help Platform for Tinnitus: A Cross-Sectional Study
Source: J Clin Med. 2026 May 23;15(11):4043. doi: 10.3390/jcm15114043 (PMC13258435; doi:10.3390/jcm15114043)
Supplement: Supplementary file 1 [file jcm-15-04043-s001.zip › jcm-4221027-supplementary.pdf]

Supplemental Figure S1

Average Time to Complete the Survey

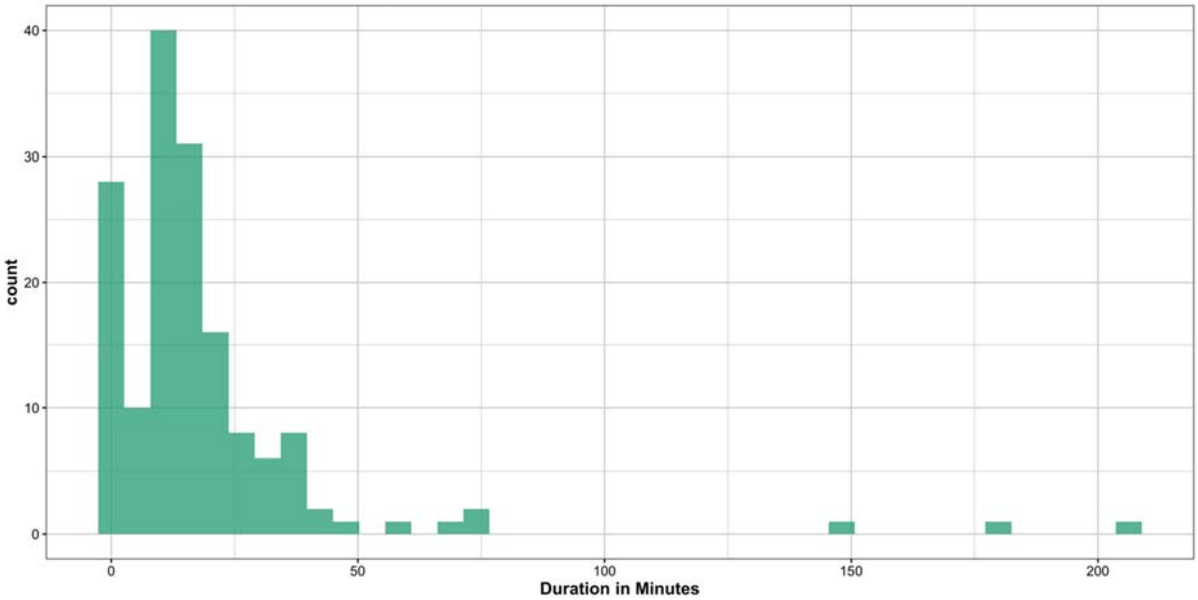

Supplemental Figure S2

Country of Residence of Participants

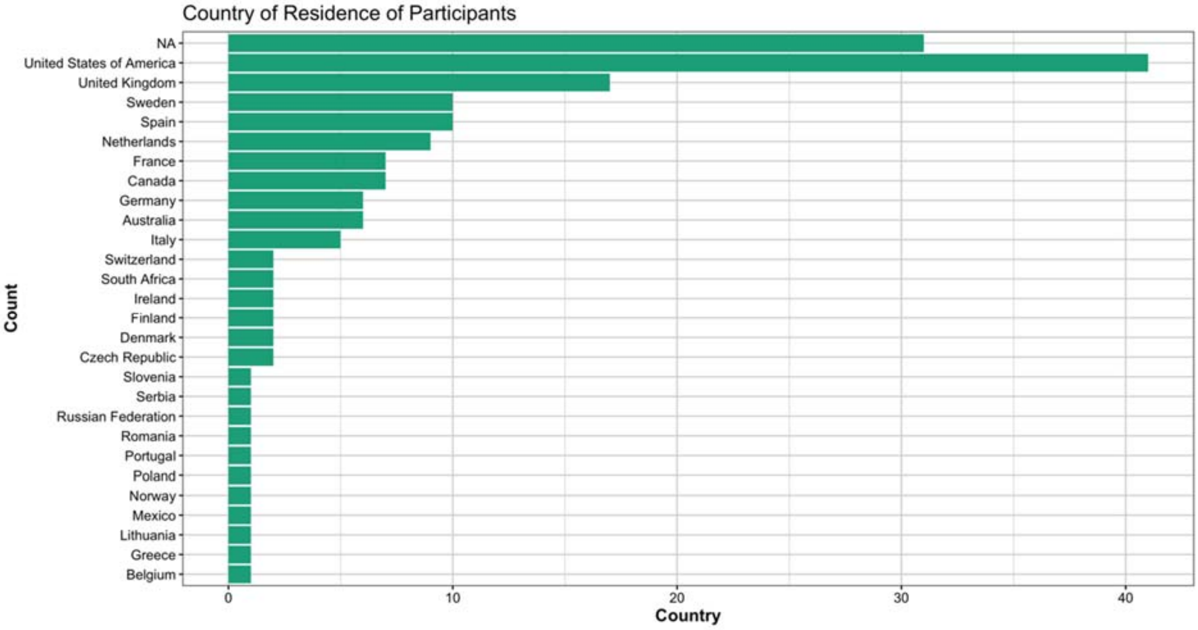

Note: NA = No Answer

Supplemental Figure S3

Centrality Indices of Network Nodes from Figure 1b

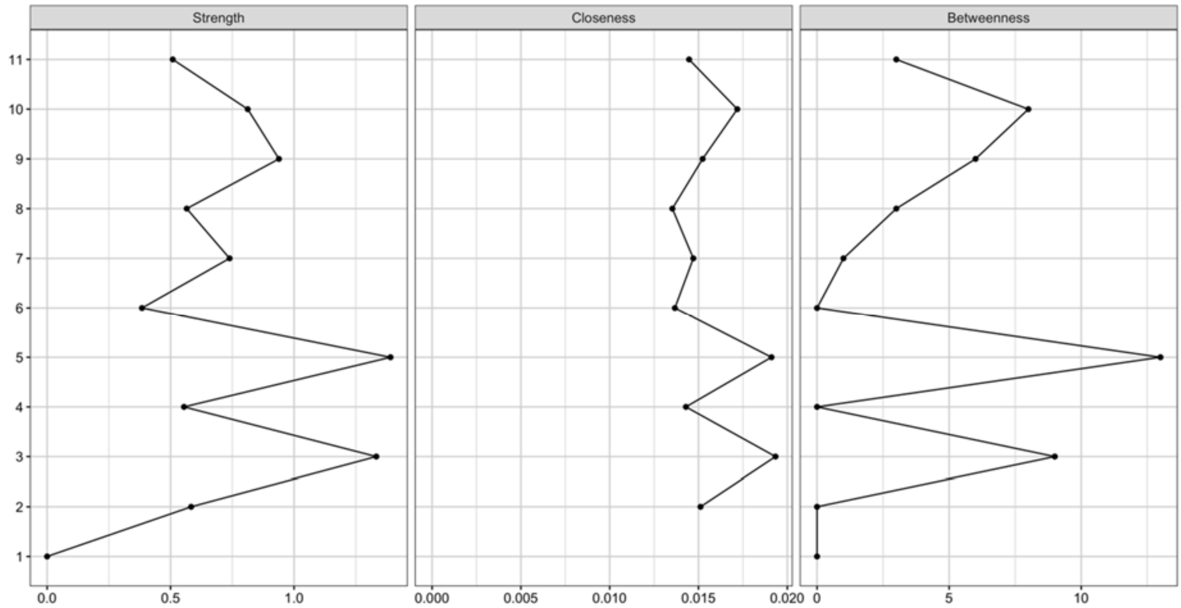

Note: This figure presents the centrality indices—Strength, Closeness, and Betweenness—of nodes in the fitted network model. Each panel corresponds to one centrality metric, with nodes, ordered according to the nodes in Figure 3B, ranked along the y-axis.

Supplemental Figure S4

Edge Weights and Bootstrap Estimates for Network Stability

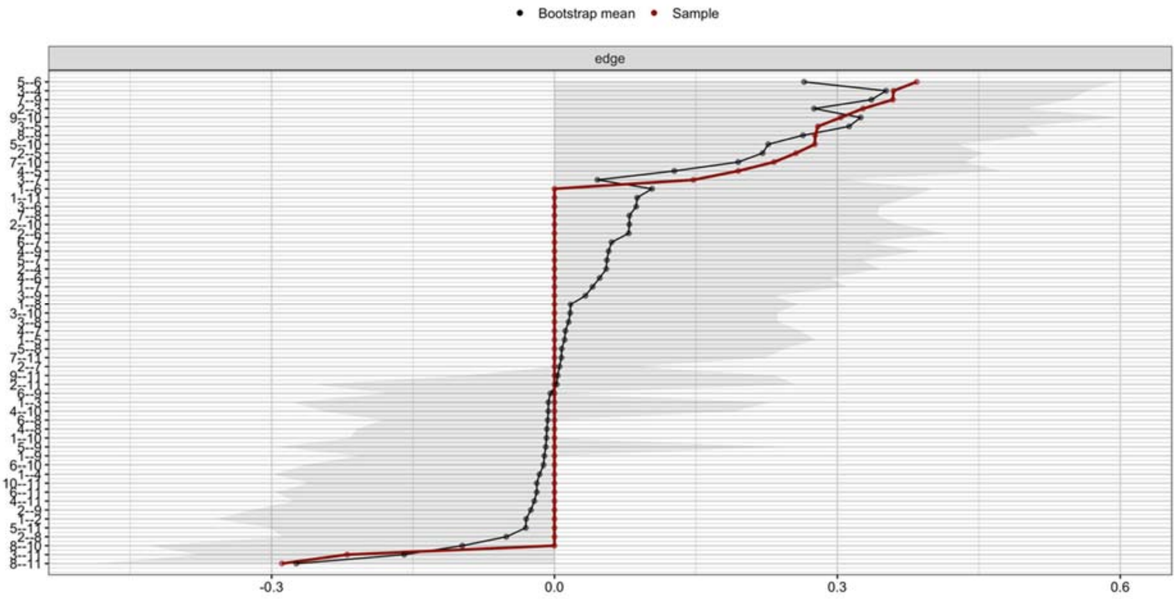

Note: This figure compares the original edge weights (in red) with bootstrap mean estimates (in black) in the network model. The y-axis represents the node pairs (numbered as per Figure 1b and the x-axis represents the edge weights, where higher values indicate stronger connections between nodes. Alignment between bootstrap means and the sample values suggests stability and reliability of the estimated edges. Discrepancies between the bootstrap and sample lines could indicate variability or uncertainty in the edge estimates.

Supplemental Table S1

STROBE Checklist

	Item No	Recommendation	Page No.
**Title and abstract**	1	(*a*) Indicate the study's design with a commonly used term in the title or the abstract	P1
		(*b*) Provide in the abstract an informative and balanced summary of what was done and what was found	P2
**Introduction**			
Background/rationale	2	Explain the scientific background and rationale for the investigation being reported	P3-4
Objectives	3	State specific objectives, including any prespecified hypotheses	P4
**Methods**			
Study design	4	Present key elements of study design early in the paper	P5
Setting	5	Describe the setting, locations, and relevant dates, including periods of recruitment, exposure, follow-up, and data collection	P5
Participants	6	(*a*) *Cohort study*—Give the eligibility criteria, and the sources and methods of selection of participants. Describe methods of follow-up<br>*Case-control study*—Give the eligibility criteria, and the sources and methods of case ascertainment and control selection. Give the rationale for the choice of cases and controls<br>*Cross-sectional study*—Give the eligibility criteria, and the sources and methods of selection of participants	P5
		(*b*) *Cohort study*—For matched studies, give matching criteria and number of exposed and unexposed<br>*Case-control study*—For matched studies, give matching criteria and the number of controls per case	
Variables	7	Clearly define all outcomes, exposures, predictors, potential confounders, and effect modifiers. Give diagnostic criteria, if applicable	
Data sources/ measurement	8*	For each variable of interest, give sources of data and details of methods of assessment (measurement). Describe comparability of assessment methods if there is more than one group	P6-7
Bias	9	Describe any efforts to address potential sources of bias	-
Study size	10	Explain how the study size was arrived at	P5

Quantitative variables	11	Explain how quantitative variables were handled in the analyses. If applicable, describe which groupings were chosen and why	
Statistical methods	12	(*a*) Describe all statistical methods, including those used to control for confounding	P8
		(*b*) Describe any methods used to examine subgroups and interactions	
		(*c*) Explain how missing data were addressed	P8
		(*d*) *Cohort study*—If applicable, explain how loss to follow-up was addressed<br>*Case-control study*—If applicable, explain how matching of cases and controls was addressed<br>*Cross-sectional study*—If applicable, describe analytical methods taking account of sampling strategy	
		(*e*) Describe any sensitivity analyses	
**Results**			
Participants	13*	(a) Report numbers of individuals at each stage of study—eg numbers potentially eligible, examined for eligibility, confirmed eligible, included in the study, completing follow-up, and analysed	P9
		(b) Give reasons for non-participation at each stage	-
		(c) Consider use of a flow diagram	-
Descriptive data	14*	(a) Give characteristics of study participants (eg demographic, clinical, social) and information on exposures and potential confounders	P9-10
		(b) Indicate number of participants with missing data for each variable of interest	P9-10
		(c) *Cohort study*—Summarise follow-up time (eg, average and total amount)	
Outcome data	15*	*Cohort study*—Report numbers of outcome events or summary measures over time	
		Case-control study—Report numbers in each exposure category, or summary measures of exposure	
		Cross-sectional study—Report numbers of outcome events or summary measures	P9-12
Main results	16	(*a*) Give unadjusted estimates and, if applicable, confounder-adjusted estimates and their precision (eg, 95% confidence interval). Make clear which confounders were adjusted for and why they were included	
		(*b*) Report category boundaries when continuous variables were categorized	
		(*c*) If relevant, consider translating estimates of relative risk into absolute risk for a meaningful time period	
Other analyses	17	Report other analyses done—eg analyses of subgroups and interactions, and sensitivity analyses	P13-19

**Discussion**			
Key results	18	Summarise key results with reference to study objectives	P20-22
Limitations	19	Discuss limitations of the study, taking into account sources of potential bias or imprecision. Discuss both direction and magnitude of any potential bias	P22
Interpretation	20	Give a cautious overall interpretation of results considering objectives, limitations, multiplicity of analyses, results from similar studies, and other relevant evidence	P20-23
Generalisability	21	Discuss the generalisability (external validity) of the study results	P22
Other information			
Funding	22	Give the source of funding and the role of the funders for the present study and, if applicable, for the original study on which the present article is based	P27
